# Genetics and Epigenetics of Manganese Toxicity

**DOI:** 10.1007/s40572-022-00384-2

**Published:** 2022-11-10

**Authors:** Sabrina Lindner, Roberto Lucchini, Karin Broberg

**Affiliations:** 1 Division of Occupational and Environmental Medicine, Department of Laboratory Medicine, Lund University, Lund, Sweden; 2 Occupational and Environmental Medicine, University of Brescia, Brescia, Italy; 3 School of Public Health, Florida International University, Miami, FL USA; 4 Institute of Environmental Medicine, Karolinska Institutet, Stockholm, Sweden

**Keywords:** Manganese, DNA methylation, SLC30A10, SLC39A8, HFE

## Abstract

**Purpose of Review:**

At elevated levels, the essential element manganese (Mn) is neurotoxic and increasing evidence indicates that environmental Mn exposure early in life negatively affects neurodevelopment. In this review, we describe how underlying genetics may confer susceptibility to elevated Mn concentrations and how the epigenetic effects of Mn may explain the association between Mn exposure early in life and its toxic effects later in life.

**Recent Findings:**

Common polymorphisms in the Mn transporter genes *SLC30A10* and *SLC39A8* seem to have a large impact on intracellular Mn levels and, in turn, neurotoxicity. Genetic variation in iron regulatory genes may to lesser extent also influence Mn levels and toxicity. Recent studies on Mn and epigenetic mechanisms indicate that Mn-related changes in DNA methylation occur early in life. One human and two animal studies found persistent changes from in utero exposure to Mn but whether these changes have functional effects remains unknown.

**Summary:**

Genetics seems to play a major role in susceptibility to Mn toxicity and should therefore be considered in risk assessment. Mn appears to interfere with epigenetic processes, potentially leading to persistent changes in developmental programming, which warrants further study.

## Introduction

Manganese (Mn) is an essential element for living organisms, including humans. It is a required cofactor for many enzymes that have critical functions in diverse processes such as forming cartilage and bone, excreting waste via the urea cycle, maintaining mitochondria, antioxidant defenses, producing glucose, brain development, and wound healing [1]. Humans mainly get Mn from dietary intake and Mn deficiency is very rare. However, excess Mn causes severe deleterious health effects in humans. These effects are observed especially in the central nervous system, since Mn accumulates in the brain [2, 3]. Mn exposure was first associated with adverse health outcomes in adults, including Mn-induced Parkinsonism and other neurodegenerative conditions, due to occupational exposures from mining, battery production, welding, and ferromanganese alloy plants [2, 4, 5]. Environmental Mn exposure has become a public health concern in recent years due to emerging evidence that children may be exposed to harmful levels of Mn from multiple sources, including drinking water, soil and dust, and possibly their diet [1]. Epidemiological studies have shown that elevated Mn exposure is associated with reductions in full scale IQ, along with adverse behavior and fine motor function in children and adolescents [6–9]; however, others have found no adverse association [10, 11]. Mechanisms linking Mn exposure to neurodevelopmental outcomes include oxidative stress, mitochondrial dysfunction, endoplasmic reticulum stress, apoptosis, neuroinflammation, and interference with neurotransmitter metabolism [12]. Recent studies have reported Mn-related alterations in the epigenetic regulation of gene expression, indicating that Mn can target the programming of cells and tissues. Epigenetic alterations may be long-term and of importance for neurodevelopment and vulnerability to brain disorders [13, 14].

Examining susceptibility factors can provide insights on the mechanisms of toxicity. One susceptibility factor for Mn toxicity is sex. Several studies have shown different associations between Mn exposure and neurological effects between girls and boys, suggesting that there could be sex-related differences in Mn sensitivity [15–17]. Another susceptibility factor is low iron (Fe) stores. Mn and Fe compete for the same protein, divalent metal transporter 1 (DMT1) [18] and blood Mn and Fe levels are therefore often inversely correlated [19]. Recent data suggest that underlying genetics is also a susceptibility factor.

In this review, we provide an overview of the genetic factors of Mn metabolism and toxicity. Further, we review epigenetic effects of early life and adult exposure to Mn and hypothesize those effects are persistent when occurring early in life.

## Genetics for Manganese Susceptibility

### Manganese Transporters

**Fig. 1:**
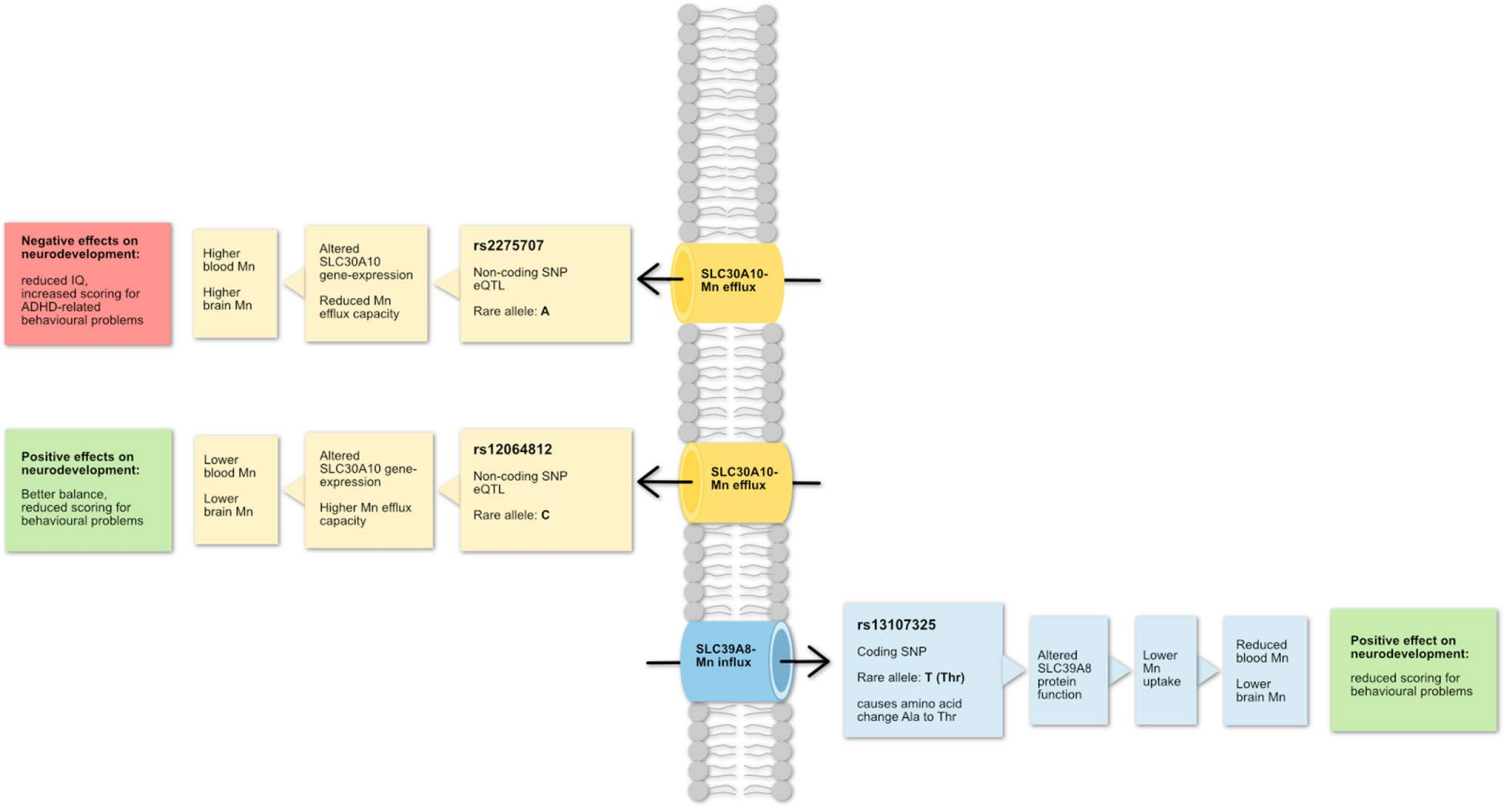
The figure summarizes results of different studies about polymorphisms in manganese (Mn) efflux transporter SLC30A10 and influx transporter SLC39A8. It shows the regulation of manganese and neurodevelopmental effects of each SNPs. Mn, manganese; SNP, single nucleotide polymorphism; ADHD, attention hyperactivity disorder

Rare variants of genes involved in Mn homeostasis can result in increased intracellular Mn levels. During the last 10 years, identification of inherited Mn transportopathies has highlighted a network of solute carrier transporters that are required for Mn homeostasis in humans. Solute carrier family 30 member 10 (SLC30A10) and solute carrier family 39 member 14 (SLC39A14) act in conjunction to excrete Mn into the bile and intestine (Fig. 1). SLC30A10 is a Mn efflux transporter, which transports Mn from the cytosol to the cell exterior and protects against Mn toxicity [20••]. SLC39A14 is a divalent metal efflux transporter, which transports zinc, Mn, Fe, and cadmium [21••]. Rare homozygous loss-of-function mutations in either gene result in Mn accumulation, even in the absence of external Mn exposure, in the basal ganglia, particularly the globus pallidus, causing Mn neurotoxicity and progressive dystonia-Parkinsonism [21••, 22••, 23••]. By contrast, SLC39A8 is the key transporter required for systemic Mn uptake and *SLC39A8* mutations lead to Mn deficiency characterized by impaired glycosylation and mitochondrial function [24••].

High-penetrance mutations associated with disease confer a high absolute risk irrespective of environmental factors and are generally rare, occurring at frequencies lower than 1% in the population. More common genetic variants in *SLC30A10* (non-coding rs2275707, rs1776029, rs12064812; Table 1) and *SLC39A8* (rs13107325, A391T) influence Mn concentrations in healthy individuals from various populations and age groups (genome-wide association study in [25•, 26, 27]), and despite being common alleles, they have a substantial effect on Mn concentrations. For example, studies have attributed a difference of up to 40% in blood Mn levels to a *SLC30A10* allele [26, 28•]. By contrast, alleles of the major arsenic methylating gene *arsenite methyltransferase* (*AS3MT*) only explain 7% of the variation in the urinary fraction of dimethylated arsenic, a metabolite involved in arsenic excretion [29]. Mendelian randomization analysis also showed that the same polymorphisms in *SLC30A10* and *SLC39A8* were associated with neurodevelopmental outcomes, particularly test scores for ADHD-related behavioral problems [28•] and contributed to differences in Mn sensitivity, particularly in girls [30]. The rs2275707 and rs12064812 variants of *SLC30A10* are classified as functional as they are expression quantitative trait loci (eQTL, GTEx database) that show lower and higher expression, respectively, in parts of the basal ganglia where Mn accumulates. Furthermore, rs2275707 was associated with significant differences in expression in blood where the variant allele correlated with lower gene expression [26].

**Table 1:** Human polymorphisms and association with manganese (Mn) levels and disease outcomes

Gene Chromosome	Major protein function^1^	Gene expression^1^	Polymorphism (alleles)^2^ Trivial name	Sequence context and functional effect of minor allele	MAF (%)^3^	Associations between genotype and manganese concentrations	Association between genotype and Mn-related outcome/comments model
Solute carrier family 30 member 10 ***SLC30A10*** Chr. 1	Mn efflux transporter [31••]	High expression in duodenum, liver, intestine, and brain	rs1776029 (G/**A**) [in strong linkage to rs2275707 (**C**/A)]	Non-coding upstream variant; eQTL^4^	1 to 21	AA ↑ blood Mn GG and GA ↓ blood Mn Prenatal dentine: AA ↓ Mn GG and GA ↑ Mn Postnatal dentine: AA ↑ Mn GG and GA ↓ Mn Early childhood dentine: AA ↑ Mn GG and GA ↓ Mn	*SLC30A10* gene-expression ↓ Mn concentration ↑ Scores for behavioral problems ↑ Sway velocity ↑ [26]/analysis adjusted for iron status, age, and gender. Zinc levels did not influence the results
			rs12064812 (T/**C**)	Non-coding variant, eQTL	10 to 38	CC ↓ blood Mn TT and TC ↑ blood Mn Prenatal dentine: CC ↑ Mn, TT and CT ↓ Mn Postnatal dentine: CC ↓ Mn TT and CT ↑ Mn Early childhood dentine: CC ↓ Mn TT and CT ↑ Mn	*SLC30A10* gene-expression ↑ Mn concentration ↓ Scores for behavioral problems ↓ Finger tapping speed and balance ↑ [26]/analysis adjusted for iron status, age, and gender. Zinc levels did not influence the results
Solute carrier family 39 member 8 ***SLC39A8*** Chr. 4	Mn influx transporter [32]	High expression in lung, liver, kidney, salivary glands, placenta, nasal epithelium, and olfactory mucosa	rs13107325 (C/**T**) A391T	Missense variant, alanine to threonine substitution, which reduces *SLC39A8* transport function, eQTL	0 to 8	CT/TT ↑ blood Mn CC ↓ blood Mn Prenatal dentine: CT/TT ↓ Mn CC ↑ Mn Postnatal dentine: CT/TT ↑ Mn CC ↓ Mn Early childhood dentine: CT/TT ↑ Mn CC ↓ Mn	Mn concentration ↓ Scores for behavioral problems ↓ [26]/analysis adjusted for iron status, age, and gender. Zinc levels did not influence the results
Homeostatic iron regulator ***HFE*** Chr. 6	Membrane protein which regulates iron homeostasis	High expression in thyroid, gall bladder, fibroblasts, adrenal gland, spleen, prostate, and uterus	rs1800562 (**G**/A) C282Y	Missense variant, tyrosine to cysteine substitution, which causes impaired function of *HFE* and higher serum iron levels [33, 34] eQTL	0 to 4	AG and GG ↓ blood Mn AA ↑ blood Mn [35]	Risk of ALS, Alzheimer’s disease ↓/only significant when both *HFE* polymorphisms were considered. Models adjusted for hemoglobin at 1-month postpartum and gestational age
			rs1799945 (**C**/G/T) H63D	Missense variant, histidine to aspartic acid substitution, which alters the tertiary structure of *HFE* and its effect on iron homeostasis [33] eQTL	1 to 17	CC and CG ↓ blood Mn GG ↑ blood Mn [35]	Mn efflux from the brain ↑ Oxidative stress in brain ↓ Susceptibility to Mn-associated neurotoxicity ↓/only significant when both *HFE* polymorphisms were considered. Models adjusted for hemoglobin at 1-month postpartum and gestational age
Transferrin ***TF*** **Chr. 3**	Iron transport protein that delivers iron from GI tract to tissues [36]	High expression in brain, liver, and salivary glands	rs1049296 (**C**/G/T) P570S	Missense variant, proline to serine substitution, associated with differential binding of iron to transferrin [35] eQTL	6 to 26	CC and CT ↑ blood Mn TT ↓ blood Mn	No significant association in the statistical analysis. Models adjusted for hemoglobin at 1-month postpartum and gestational age
ATPase cation transporting 13A2 ***ATP13A2*** Chr. 1	Membrane P5-type ATPase pump which transports polyamines; mutations cause juvenile Parkinson’s disease	High expression in brain, spleen, salivary gland, and lungs	rs4920608 (C/**T**)	Intronic variant, eQTL	26 to 61		Mn toxicity ↑ Protection against Parkinsonian changes ↓ [37] With increasing Mn exposure, motor coordination in CT and CC ↓ [38]/analysis adjusted for age and gender. Smoking and alcohol consumption did not influence the results
			rs2871776 (**A**/G)	Intronic variant, eQTL, associated with destroying binding site for transcription factor INSM1	25 to 51		With increasing Mn exposure, motor coordination in GG and GA ↓ [38]/analysis adjusted for age and gender. Smoking and alcohol consumption did not influence the results
(Manganese) superoxide dismutase 2 ***SOD2*** Chr. 6	Mn-dependent antioxidant protein which converts superoxide byproducts of oxidative phosphorylation to hydrogen peroxide and oxygen	High expression in musculoskeletal system, appendix, lung, bladder, and liver	rs2758352 (**A**/C/G/T)	Intronic variant, eQTL, role in scavenging ROS^5^ [39]	13 to 41	AA and AG ↑ serum Mn GG ↓ serum Mn	Risk of high Mn levels and for spontaneous preterm birth in AA and AG ↑ Interaction effect between maternal Mn level and *SOD2* in the risk of SPB^6^ ↑ [39]/adjusted for sampling time, maternal age, BMI, education, occupation, parity, spontaneous abortion history, folic acid use, medication use, passive smoking, and child gender
(Extracellular) superoxide dismutase 3 ***SOD3*** Chr. 4	Antioxidant protein that scavenges ROS	High expression in fat, prostate, kidney, lungs	rs699473 (C/**T**)	Intronic variant, role in scavenging ROS [39] eQTL	25 to 63	CT and TT ↓ serum Mn CC ↑ serum Mn	Risk of high Mn level and for spontaneous preterm birth in CC ↑ [39]/adjusted for sampling time, maternal age, BMI, education, occupation, parity, spontaneous abortion history, folic acid use, medication use, passive smoking, and child gender
Catalase ***CAT*** Chr. 11	Antioxidant enzyme that scavenges ROS	High expression in liver, fat, kidney, and lungs	rs769214 (**G**/A)	Upstream gene variant, eQTL, role in scavenging ROS [39]	33 to 73	GG ↑ serum Mn AA/AG ↓ serum Mn	Risk for spontaneous preterm birth in GG ↑ [39]/adjusted for sampling time, maternal age, BMI, education, occupation, parity, spontaneous abortion history, folic acid use, medication use, passive smoking, and child gender
Glutathione S-transferase theta 1 ***GSTT1*** Chr. 22	Catalyzes the conjugation of reduced glutathione with electrophilic and hydrophilic compounds	High expression in liver, brain, duodenum, and prostate	(wt/del)^7^	Deletion causes loss of function		Children with autism spectrum disorder with *GSTT1* deletion had increased odds of having higher Mn in blood [40]	Adjusted for socioeconomic status. Range of the *GSTT1* deletion frequency in worldwide populations: 11.6 to 51.6% [41]
Apolipoprotein E ***APOE*** Chr. 19	Lipid binding plasma glycoprotein, important for general and neuronal lipid metabolism by directing lipid transfer, uptake, and excretion [42]	High expression in liver, kidney, adrenal, fat, and brain	rs429358 (T/**C**)	Missense variant, cysteine to arginine substitution, eQTL	9 to 27	No association between *apolipoprotein E* polymorphism ε4 and Mn concentrations in mothers and newborns [43]	No association/adjusted for parity, BMI, age, seafood intake, and ever-smoking
			rs7412 (C/**T**)	Missense variant, arginine to cysteine substitution, eQTL	4 to 10	No association between *apolipoprotein E* polymorphism ε4 and Mn concentrations in mothers and newborns [43]	No association/adjusted for parity, BMI, age, seafood intake, and ever-smoking

^1^Information taken from www.ncbi.nlm.nih.gov/gene, www.ensembl.org/Homo_sapiens/Gene, and gtexportal.org/home/gene/

^2^Rare alleles written in bold

^3^Minor allele frequency (MAF %) shows the range of frequency in five groups of populations (African, American, East Asian, European, South Asian) provided in ensembl.org/Homo_sapiens/Variation/Population

^4^*eQTL*, expression quantitative trait loci

^5^*SPB*, spontaneous preterm birth

^6^*ROS*, reactive oxygen species

^7^*wt/del*, wildtype/deletion

Further support for the effect of *SLC39A8* rs13107325 (which encodes an Ala-391-Thr in the SLC39A8 protein) on Mn regulation comes from a recent animal and human study [44]. CRISPR/Cas9-mediated knock-in was used to generate a mouse model carrying the SLC39A8 amino acid substitution (Ala-391-Thr) variant and mice carrying this variant had lower blood Mn levels than mice carrying the Ala variant. These mice lines exhibited tissue-specific abnormalities in Mn homeostasis, with decreases in liver and kidney Mn levels and increased biliary Mn levels, providing in vivo evidence of altered transporter function. SLC39A8 391-Thr was also associated with reduced triantennary plasma N-glycan species in a population-based human cohort. The *SLC39A8* rs13107325 variant is one of the most pleiotropic polymorphisms known so far and has been repeatedly associated with neurological and metabolic disorders [45, 46]. Interestingly, *SLC39A8* polymorphisms, including rs13107325, and polymorphisms in linkage disequilibrium with rs13107325, are associated with different magnetic resonance imaging phenotypes of the brain [47].

So far, no studies have reported associations between common polymorphisms in the Mn transporter gene *SLC39A14* and Mn levels or Mn toxicity.

### Iron Transporters

Earlier studies investigated the role of genes involved in Fe metabolism in Mn susceptibility because of the well-established inverse correlation between Fe stores and Mn absorption [19]. Indeed, Fe and Mn likely share some transporters and regulatory proteins. Type 1 hereditary hemochromatosis is caused by being a homozygous carrier of missense mutations (His-63-Asp or Cys-282-Tyr) in the *homeostatic iron regulator* (*HFE*) gene; these mutations lead to increased Fe uptake from the gastrointestinal tract. Mice carrying the His-67-Asp *Hfe* mutation, which is homologous to the His-63-Asp mutation in humans, had lower Mn levels in the blood, liver, and brain after Mn inhalation, and lower toxicity of inhaled Mn [33]. Similar results were found when analyzing Mn levels in *Hfe*^−/−^ and *Hfe*^+/+^ mice, revealing that *Hfe*^−/−^ mice had lower Mn and higher Fe concentrations [35]. A pilot study of 141 human individuals living near a ferro-Mn refinery in the USA only detected a significant association between hair Mn levels and estimated ambient air Mn levels when polymorphisms in both *HFE* and the Fe storage gene *transferrin* (*TF*) were included in linear models, but not with either gene alone [48] (Table 1). Furthermore, among 332 pregnant Mexican women exposed to Mn from the environment, heterozygous carriers of either of the *HFE* polymorphisms (Cys-282-Tyr or His-63-Asp) had 12% lower blood Mn levels than women with no *HFE* variants [35].

### Other Genes

In the juvenile form of Parkinsonism, mutations are found in *ATPase cation transporting 13A2I* (*ATP13A2*), which encodes a P5-type ATPase pump recently shown to transport polyamines [49]. Polymorphisms in *ATP13A2* significantly modified the effects of Mn exposure on motor coordination in elderly people in Italy [38] (Table 1).

The possible consequences of Mn exposure early in life have been explored in the context of birth outcomes and underlying genetics. In a Chinese nested case–control study, higher maternal (collected in gestational weeks 4–22) serum Mn concentrations were associated with preterm birth (before week 37), and this association was modified by the genotype of genes encoding antioxidant proteins including superoxide dismutases (*SOD2* and *SOD3*) and catalase (*CAT*) [39].

Further, Rahbar et al. [40] evaluated 266 age- and sex-matched pairs of Jamaican children with autism spectrum disorder and normally developing controls (2–8 years) to determine whether copy number variation of the xenobiotic metabolizing gene *glutathione S-transferase theta 1* (*GSTT1*) modifies the association between blood Mn concentrations and autism spectrum disorder. They found a significant interaction between *GSTT1* copy number and blood Mn concentrations: compared to controls, autism spectrum disorder cases with *GSTT1* homozygous for deletion of the gene on both chromosomes had 4.35 times higher odds of blood Mn concentrations above 12 μg/l vs. below 8.3 μg/l. However, the confidence interval was very wide.

Trdin et al. [43] did not find any association between *apolipoprotein E* polymorphism ε4 and Mn concentrations in mothers and newborns.

## Epigenetic Effects of Manganese

Epigenetic changes are heritable changes in gene expression and regulation that are not coded by the DNA sequence, but by various modifications of the DNA. For example, DNA methylation, the specific methylation of cytosine residues directly upstream of a guanine, is essential for embryogenesis and for the maintenance of cell lineage-specific gene expression throughout life [50]. Transcript levels may also be regulated by non-coding RNAs, such as microRNAs, which regulate gene expression post-transcriptionally by hybridizing to messenger RNAs, leading to translational repression or degradation of the target RNA [51]. Another regulatory mechanism of gene expression entails histone modifications, which affect chromatin structure; an open chromatin structure facilitates active transcription, while a closed structure limits transcription. However, the histone-based “epigenetic code” has recently been challenged [52].

The pre- and postnatal environments are important determinants of disease susceptibility later in life [53] and this influence is thought to be mediated mainly through alterations in DNA methylation, which subsequently alter the epigenetic programming of the child and can lead to long-term negative health outcomes. Epigenetic modification by external factors was clearly demonstrated for smoking [54]. Increasing evidence also shows that early-life metal exposure may modulate the epigenetic landscape (e.g., as shown for methylmercury in [55] and for arsenic in [56]).

The symptoms of hypermanganesemia syndromes are partially reversible, i.e., the Mn load and disease progression can be ameliorated in carriers of loss-of-function mutations in *SLC30A10* and *SLC39A14* with chelation therapy together with Fe supplementation [57, 58]. However, we do not know the long-term effects of external Mn exposure and whether Mn changes cellular programming, such as via epigenetic modifications, also remains unclear. Additional research will also be needed to test the influence of Mn on DNA methylation and whether epigenetic factors change the individual’s predisposition to Mn toxicity. However, increasing evidence suggests that Mn targets the epigenetic machinery, by a yet-unknown mechanism. Below, we summarize several studies in humans and animals that explore the effect of Mn on epigenetic factors.

Only one study has, to our knowledge, evaluated Mn exposure in relation to histone modifications. In a cross-sectional study of steel workers, estimated air metal concentrations were correlated with histone modification in blood leukocytes [59] (Table 2). However, no association was found between Mn concentration in air (mean 11.26 μg/m^3^ SD ± 30.41) and histone modifications.

**Table 2:** Human epidemiological studies of manganese (Mn) exposure and DNA methylation

Study and study type	Gene Chromosome	Major protein/RNA function^1^	Gene expression^1^	CG methylation	Associations between Mn and on DNA methylation (DNAm)/adjustments
Nwanaji-Enwerem et al. [60] Longitudinal cohort study of aging	PhenoAge, epigenetic clock of DNA methylation in multiple genes	PhenoAge predictor of lifespan, physical functioning, and healthspan [34]	n.a	Calculated using methylation of 513 CpGs	Higher urine Mn concentration associated with higher PhenoAge/adjusted for chronological age, season of visit, GFR, BMI, alcohol intake, pack-years, smoking status, education, white blood cell proportion, and in sensitivity analysis diabetes, hypertension, and ischemic heart disease
Kresovich et al. [61] Longitudinal cohort study of aging	Interleukin 1β ***IL-1β*** Chr. 2	Involved in inflammatory response, cell proliferation, differentiation, and apoptosis	High expression in bone marrow, appendix, and urinary bladder	n.a.^2, 3^	No association Observing positive linear trends between estimated diary Mn intake and interleukin proteins/adjusted for age, race, BMI, education, smoking status, alcohol intake, total caloric intake, total dietary intakes of calcium and magnesium, blood cell composition, and DNA processing batch
	Interleukin 6 ***IL-6*** Chr. 7	Involved in acute and chronic inflammation and maturation of B cells	High expression in urinary bladder, gall bladder, and lung	n.a	No association/for adjustments see above
	C-X-C motif chemokine ligand 8 ***CXCL8 [IL-8]*** Chr. 4	Involved in inflammatory response, secreted by i.a. mononuclear macrophages and neutrophils; chemotactic factor by guiding neutrophils to the site of infection, participates in the proinflammatory signaling cascade	High expression in bone marrow, appendix, urinary bladder, and gall bladder	n.a	No association
	Tumor necrosis factor ***TNF*** Chr. 6	Multifunctional proinflammatory cytokine, mainly secreted by macrophages, involved in regulation of cell proliferation, differentiation, apoptosis	High expression in bone marrow, lymph node, and lung	n.a	No association
	Vascular endothelial growth factor ***VEGFA*** Chr. 6	Heparin-binding protein, induces proliferation and migration of vascular endothelial cells, essential for physiological and pathological angiogenesis, upregulated in many tumors	High expression in thyroid, prostate, lung, endometrium, and gall bladder	n.a	No association
	TNF receptor superfamily member 1B ***TNFRSF1B*** Chr. 1	Protein of the TNF-receptor superfamily, forms a heterocomplex with TNF-receptor 1 and mediates the recruitment of two anti-apoptotic proteins (c-IAP 1 and c-IAP2)	High expression in appendix, spleen, placenta, lymph node, and bone marrow	n.a	No association
	C-reactive protein ***CRP*** Chr. 1	Involved in complement activation and amplification, involved in several host defense related functions based on ability to recognize foreign pathogens and damaged cells	High expression in liver and gall bladder	n.a	No association
	Intercellular adhesion molecule 1 ***ICAM1*** Chr. 19	Cell surface glycoprotein, binds to integrins of type CD11a/CD18, or CD11b/CD18, exploited by rhinovirus as a receptor	High expression in lung, bone marrow, gall bladder, and liver	n.a	No association
	Vascular cell adhesion molecule 1 ***VCAM1*** Chr. 1	Sialoglycoprotein expressed by cytokine-activated endothelium, mediates leukocyte-endothelial cell adhesion and signal transduction	High expression in spleen, lymph node, and kidney	n.a	No association
	NFKB activating protein ***NKAP*** Chr. X	Activation of transcription factor NF-κβ	High expression in placenta, adrenal, ovary, lymph node, and other organs	n.a	Higher Mn estimated intake^4^ associated with higher DNAm in non-promoter region; non-significant after adj. for multiple comparisons
	NFKB activating protein pseudogene 1 ***NKAPP1*** Chr. X	NF-κβ activating protein pseudogene, associated with histone deacetylase HDAC3 and the Notch corepressor complex, transcriptional repressor of Notch target genes	High expression in ovary, bone marrow, and thyroid	n.a	Higher Mn estimated intake associated with higher DNAm in non-promoter region; non-significant after adj. for multiple comparisons
Bozack et al. [62••] Mother–child cohort	RNA binding fox-1 homolog 1 ***RBFOX1 (A2BP1)*** Chr. 16	Binds RNA and is involved in tissue-specific pre-mRNA splicing of transcripts in neuronal development	High expression in brain and heart	cg02042823	Higher maternal Ery^5^-Mn associated with increased DNAm in cord blood and mid-childhood blood in all infants, and in male infants/adjusted for infant sex, race/ethnicity, gestational age, nulliparous, maternal age at enrollment, pre-pregnancy BMI, education, smoking, household income, and estimated cord blood cell-type proportions
	Leucine rich repeat containing 47 ***LRRC47*** Chr. 1	Enables RNA binding activity, predicted to be involved in phenylalanyl-tRNA aminoacylation	High expression in brain, prostate, ovary, and other organs	cg00954161	Higher maternal Ery-Mn associated with increased DNAm in cord blood and mid-childhood blood in female infants
	Succinate-CoA ligase GDP-forming subunit beta ***SUCLG2*** Chr. 3	GTP-specific beta subunit of succinyl-CoA synthetase, catalyzes the reaction of formation of succinyl-CoA and succinate	High expression in colon, kidney, small intestine, and liver	cg11161853	Higher maternal Ery-Mn associated with lower DNAm in cord blood in female infants
	LDL receptor related protein associated protein 1 ***LRPAP1*** Chr. 4	Interacts with the low-density lipoprotein receptor-related protein, homozygous mutation in this gene causes myopia 23	High expression in kidney, placenta, testis, and heart	cg23903787	Higher maternal Ery-Mn associated with increased DNAm in cord blood and mid-childhood blood in female infants
	Neuropeptide Y receptor Y1 ***NPY1R*** Chr. 4	Transmembrane protein, mediates the function of the neurotransmitter neuropeptide Y and gastrointestinal hormone peptide YY	High expression in spleen, fat, adrenal, and kidney	cg19908812	Higher maternal Ery-Mn associated with lower DNAm in cord blood in female infants
	Mitotic arrest deficient 1 like 1 ***MAD1L1*** Chr. 7	Component of the mitotic spindle-assembly checkpoint	High expression in testis, spleen, lymph node, and fat	cg26462130	Higher maternal Ery-Mn associated with increased DNAm in cord blood and mid-childhood blood in female infants
	Intergenic	n.a	n.a	cg08904630	Higher maternal Ery-Mn associated with increased DNAm in cord blood and mid-childhood blood in female infants
	RNA binding motif single stranded interacting protein 2 ***RBMS2*** Chr. 12	Binds single-stranded DNA/RNA, involved in DNA replication, gene transcription, cell cycle progression, and apoptosis	High expression in lung, placenta, and fat	cg22799518	Higher maternal Ery-Mn associated with higher DNAm in cord blood in female infants and lower DNAm in mid-childhood blood
	Chromosome 1 open reading frame 54 ***C1orf54*** Chr. 1	Protein predicted to be located in extracellular region	High expression in spleen, lymph node, and gall bladder	cg03763518	Higher maternal Ery-Mn associated with lower DNAm in cord blood and in mid-childhood blood in males
	Intergenic	n.a	n.a	cg01744822	Higher maternal Ery-Mn associated with lower DNAm in cord blood and mid-childhood blood in female infants
	Intergenic	n.a	n.a	cg15712310	Higher maternal Ery-Mn associated with lower DNAm in cord blood and mid-childhood blood in female infants
Aung et al. [63] Pregnancy cohort	AT-rich interaction domain 2 ***ARID2*** Chr. 12	Component of chromatin remodeling protein complexes [42], role in embryonic patterning, cell lineage gene regulation, cell cycle control	High expression in testis and thyroid	cg01183821	Maternal blood-Mn associated with increased maternal blood DNAm at CpG site near to gene *ARID2*/adjusted for cell type principal components, maternal age, fetal sex, and hybridization date
Maccani et al. [64] Mother–child cohort	EMX2 opposite strand/antisense RNA ***EMX2OS*** Chr. 10	Long non-coding RNA transcript regulating the *EMX2* gene	High expression in endometrium, kidney, brain, ovary, and urinary bladder	cg16063747	Highest tertile of infant toenail Mn associated with higher placental DNAm/adjusted for maternal age, birth weight percentile, delivery method, and infant gender
	ATPase family AAA domain containing 2B ***ATAD2B*** Chr. 2	Protein belonging to AAA ATPase family, located in nucleus, partly to replication sites, consistent with a chromatin-related function	High expression in lymph node, testis, skin, appendix, and other organs	cg08192560	Lowest tertile of infant toenail Mn associated with higher placental DNAm
	FTO alpha-ketoglutarate dependent dioxygenase ***FTO*** Chr. 16	Nuclear protein of the AlkB related non-haem iron and 2-oxogluyarate oxygenase family, non-heme iron enzymes function to reverse alkylated DNA and RNA damage by oxidative demethylation	High expression in brain, adrenal, thyroid, and lung	cg26692097	Lowest tertile of infant toenail Mn associated with higher placental DNAm
	RPGRIP1 like ***RPGRIP1L*** Chr. 16	Interacts with nephrocystin-4, defects in this gene cause Joubert syndrome type 7 and Meckel syndrome type 5	High expression in testis, brain, and thyroid		
	Engrailed homeobox 1 ***EN1*** Chr. 2	Homeodomain-containing protein, implicated in control of pattern formation during development of CNS	High expression in fat, lymph node, and skin	cg07419575	High and low tertiles of infant toenail Mn associated with higher placental DNAm compared to referent tertile (medium tertile)
	Long intergenic non-protein coding RNA 908 ***LINC00908*** Chr. 18	Not known	High expression in ovary, endometrium, and testis	cg22284422	Highest tertile of infant toenail Mn associated with higher placental DNAm; significant correlation to lower birth weight [34]
Appleton et al. [65•] Mother–child cohort	Nuclear receptor subfamily 3 group C member 1 ***NR3C1*** Chr. 5	Glucocorticoid receptor, transcription factor that binds to glucocorticoid response elements to activate their transcription, or as a regulator of other transcription factors, involved in regulation of stress response	High expression in fat, lung, placenta, urinary bladder, and other organs	n.a.^6^	Highest tertile of Mn in infant toenails associated with increased DNAm in placenta in all children and in female infants/adjusted models for maternal age, race, education, pre-pregnancy BMI, prenatal tobacco use, prenatal depression, infant gender, and birthweight percentile

^1^Information taken from www.ncbi.nlm.nih.gov/gene, www.ensembl.org/Homo_sapiens/Gene, and gtexportal.org/home/gene/

^2^*n.a.*, not available

^3^Kresovich et al. [61] used average DNA methylation of CpGs in each gene

^4^Kresovich et al. [61] estimated total dietary intakes of Mn and other macro- and micronutrients using a self-administered, semi-quantitative food frequency questionnaire; responses were processed through a nutrient database to estimate usual daily nutrient intakes

^5^*Ery*, erythrocyte

^6^Appleton et al. [65•] computed mean *M*-values across the interrogated CpG regions. The *M*-value is calculated as the log2 ratio of the intensities of methylated probe vs. unmethylated probe

Nwanaji-Enwerem et al. [60] examined the relationship of urine Mn levels in elderly men (Normative Aging Study) over a 24-h period with three DNA methylation-based measures of biological aging: DNAmAge, GrimAge, and PhenoAge. Urine Mn (mean 1.4 ng/ml ± 0.4 SD) was linked to PhenoAge. A 1 ng/ml increase in urine Mn was associated with a 9.93-year increase in DNA-methylation based biological age. Because Mn is normally excreted via bile, not urine, this finding may be explained by a partial shift to excretion of Mn in urine related to kidney disease, which in turn accelerates biological aging. The study did adjust for kidney function but there may be residual confounding and further studies are warranted to clarify this finding.

In the same cohort of elderly men, estimated dietary Mn intake (categorized in quartiles from ≤ 2.68 to ≥ 5.48 mg/day) from food/beverages and supplements were correlated with circulating biomarkers of inflammation and DNA methylation of genes involved in the production of biomarkers of inflammation [61]. No strong evidence was found for increasing Mn intake and altered DNA methylation of the genes, but trends (non-significant after adjustment for multiple comparisons) were found for methylation of non-promoter CpG sites in genes encoding NF-κβ member activators.

Bozack et al. [62••] analyzed whether Mn in maternal erythrocytes (median 15.80 ng/g IQR 13.10, 19.70) during the first trimester was associated with differentially methylated positions (DMPs) and regions (DMRs) in cord blood and tested if associations persisted in blood collected in mid-childhood (6–10 years old) in a cohort of 361 children. Mn was associated with increased methylation of cg02042823 in the gene *RNA binding fox-1 homolog 1* (*RBFOX1*, also called *A2BP1*) in cord blood, and this association was still significant, but attenuated in blood collected at mid-childhood. Two and nine Mn-associated DMPs were identified in male and female infants, respectively, with two and six persisting in mid-childhood. The DMPs identified in males and females did not overlap. This finding supports that prenatal exposure to Mn may result in changes in DNA methylation that persist into childhood and that the changes may be sex-specific. In cord blood, Mn exposure was associated with a DMR annotated with *tenascin XB* (*TNXB*) in the human leukocyte antigen region, but this did not persist into childhood. In maternal blood of 97 non-smoking pregnant women, maternal Mn (geometric mean 12.67 µg/l) concentrations were non-significantly associated with hypermethylation at four DNA methylation sites, one of which was near the gene *AT-rich interaction domain 2* (*ARID2*) [63]. Genes encompassing Mn-associated methylated sites were enriched for cellular nitrogen metabolism, cell cycle process, nucleic acid metabolism, and negative regulation of response to DNA damage stimulus.

In a birth cohort, Mn concentrations measured in infant toenails were correlated with genome-wide DNA methylation in 61 placental samples [64]. The Mn levels ranged from 0.131 to 5.666 µg/g toenail where the second, or referent, tertile ranged from 0.394 to 0771 µg/g. Five significantly differentially methylated loci (annotated genes *LINC00908* (*LOC284276*), *FTO*, *EMX2OS*, *ATAD2B*, and *EN1*) reside in neurodevelopmental, fetal growth, and cancer-related genes. cg22284422, located within *LOC284276*, was associated with birth weight; for every 10% increase in methylation, lower birth weights were observed. The observations suggest a link between prenatal micronutrient levels, placental epigenetic status, and birth weight.

A cohort of healthy-term singleton pregnancies [65•] studied prenatal Mn exposure and DNA methylation in placentas, focusing on methylation of *nuclear receptor subfamily 3 group C member 1* (*NR3C1*), encoding the glucocorticoid receptor essential for the body’s stress response. Mn concentrations (median 0.56 μg/g) were measured in infant toenails, which reflect long-term external exposure at a fairly reproducible level [66]. Compared to the lowest exposure tertile, the highest tertile of Mn in toenails was associated with a small (0.80%) but significant increase in placental *NR3C1* methylation. Whether this small effect has functional consequences is unknown but there is some evidence that higher *NR3C1* methylation is the epigenetic nexus between early life stress and later life psychiatric disorders [67].

Animal studies have also been performed. Pregnant mice were treated with 800 ppm MnCl_2_ in their diet from gestational day 10 through day 21 [68]. Following 800-ppm Mn exposure, a CpG promoter microarray study found hypermethylation of the promoter regions of 24 genes in the hippocampal dentate gyrus of male offspring. After 800-ppm Mn exposure through the adult stage, hypermethylation and transcript downregulation was confirmed in *Pvalb*, *Mid1*, *Atp1a3*, and *Nr2f1*. These results suggest that Mn exposure alters epigenetic gene regulation and programming of cellular populations related to neurogenesis. Still, how the Mn dose translates to human exposure is unclear.

In a later animal study, pregnant mice were given drinking water with high concentration of Mn (MnCl_2_ of 10 mg/l in the water, to compare with the US EPA health advisory value for Mn in drinking water of 0.3 mg/l) from gestational days 1–10 and young male offspring were tested for behavioral deficits [69]. In utero exposure to Mn resulted in multiple behavioral abnormalities that persisted into adulthood. Brain samples from three Mn-treated and three control animals were evaluated for changes in the frontal cortex of CpG island methylation in promoter regions and associated changes in gene expression. In Mn-exposed animals compared to water-treated controls, the *chromodomain helicase DNA binding protein 7* (*Chd7*) gene, essential for neural crest cell migration and patterning, was found to be hypomethylated and showed higher gene expression. However, this study should be interpreted with caution, as the Mn level was very high, and the study group was small.

## Conclusions

Underlying genetics clearly plays a critical role in Mn metabolism and toxicity. The genotypes of the Mn transporter genes *SLC39A8* and *SLC30A10* have repeatedly been shown to influence Mn homeostasis and susceptibility to Mn neurotoxicity, and the association between the common variants of these genes and intracellular Mn concentrations is one of the strongest gene-environment interactions reported so far. Genes involved in Fe uptake and metabolism may modify Mn levels as well, although to a lesser extent.

The epigenetic effect of Mn is a new and growing research field. Thus far, one human study reported Mn-related changes in DNA methylation from birth to childhood. This finding suggests that prenatal exposure to Mn may result in changes in DNA methylation that persist into childhood. Still, no gene has consistently, and across studies, been found to be altered in relation to Mn exposure. DNA methylation is the predominant epigenetic factor evaluated so far and further studies on histone modification and non-coding RNA in relation to Mn are warranted.

## References

[1] Lucchini RG, Aschner M, Kim Y, Nordberg G, Costa M (2022). Manganese. Handbook on the toxicology of metals.

[2] Baker MG, Criswell SR, Racette BA, Simpson CD, Sheppard L, Checkoway H, Seixas NS (2015). Neurological outcomes associated with low-level manganese exposure in an inception cohort of asymptomatic welding trainees. Scand J Work Environ Health.

[3] Lai JC, Minski MJ, Chan AW, Leung TK, Lim L (1999). Manganese mineral interactions in brain. Neurotoxicology..

[4] Aschner M, Erikson KM, Herrero Hernández E, Tjalkens R (2009). Manganese and its role in Parkinson’s disease: from transport to neuropathology. Neuromolecular Med.

[5] Lucchini R, Apostoli P, Perrone C, Placidi D, Albini E, Migliorati P, Mergler D, Sassine MP, Palmi S, Alessio L (1999). Long-term exposure to “low levels” of manganese oxides and neurofunctional changes in ferroalloy workers. Neurotoxicology..

[6] Bouchard MF, Sauvé S, Barbeau B, Legrand M, Brodeur MÈ, Bouffard T, Limoges E, Bellinger DC, Mergler D (2011). Intellectual impairment in school-age children exposed to manganese from drinking water. Environ Health Perspect.

[7] Mora AM, Córdoba L, Cano JC, Hernandez-Bonilla D, Pardo L, Schnaas L, Smith DR, Menezes-Filho JA, Mergler D, Lindh CH, Eskenazi B, van Wendel de Joode B (2018). Prenatal mancozeb exposure, excess manganese, and neurodevelopment at 1 year of age in the Infants’ Environmental Health (ISA) study. Environ Health Perspect..

[8] Oulhote Y, Mergler D, Barbeau B, Bellinger DC, Bouffard T, Brodeur MÈ, Saint-Amour D, Legrand M, Sauvé S, Bouchard MF (2014). Neurobehavioral function in school-age children exposed to manganese in drinking water. Environ Health Perspect.

[9] Rahman SM, Kippler M, Tofail F, Bölte S, Hamadani JD, Vahter M (2017). Manganese in drinking water and cognitive abilities and behavior at 10 years of age: a prospective cohort study. Environ Health Perspect.

[10] Soler-Blasco R, Murcia M, Lozano M, González-Safont L, Amorós R, Ibarluzea J, Broberg K, Irizar A, Lopez-Espinosa MJ, Lertxundi N, Marina LS, Ballester F, Llop S (2020). Prenatal manganese exposure and neuropsychological development in early childhood in the INMA cohort. Int J Hyg Environ Health..

[11] Irizar A, Molinuevo A, Andiarena A, Jimeno-Romero A, San Román A, Broberg K, Llop S, Soler-Blasco R, Murcia M, Ballester F, Lertxundi A (2021). Prenatal manganese serum levels and neurodevelopment at 4 years of age. Environ Res..

[12] Tinkov AA, Paoliello MMB, Mazilina AN, Skalny AV, Martins AC, Voskresenskaya ON, Aaseth J, Santamaria A, Notova SV, Tsatsakis A, Lee E, Bowman AB, Aschner M (2021). Molecular targets of manganese-induced neurotoxicity: a five-year update. Int J Mol Sci.

[13] Iraola-Guzmán S, Estivill X, Rabionet R (2011). DNA methylation in neurodegenerative disorders: a missing link between genome and environment?. Clin Genet.

[14] Jaffe AE, Gao Y, Deep-Soboslay A, Tao R, Hyde TM, Weinberger DR, Kleinman JE (2016). Mapping DNA methylation across development, genotype and schizophrenia in the human frontal cortex. Nat Neurosci.

[15] Bauer JA, Claus Henn B, Austin C, Zoni S, Fedrighi C, Cagna G, Placidi D, White RF, Yang Q, Coull BA, Smith D, Lucchini RG, Wright RO, Arora M (2017). Manganese in teeth and neurobehavior: sex-specific windows of susceptibility. Environ Int..

[16] Gunier RB, Arora M, Jerrett M, Bradman A, Harley KG, Mora AM, Kogut K, Hubbard A, Austin C, Holland N, Eskenazi B (2015). Manganese in teeth and neurodevelopment in young Mexican-American children. Environ Res..

[17] Mora AM, Arora M, Harley KG, Kogut K, Parra K, Hernández-Bonilla D, Gunier RB, Bradman A, Smith DR, Eskenazi B (2015). Prenatal and postnatal manganese teeth levels and neurodevelopment at 7, 9, and 10.5 years in the CHAMACOS cohort. Environ Int..

[18] Illing AC, Shawki A, Cunningham CL, Mackenzie B (2012). Substrate profile and metal-ion selectivity of human divalent metal-ion transporter-1. J Biol Chem.

[19] Ljung KS, Kippler MJ, Goessler W, Grandér GM, Nermell BM, Vahter ME (2009). Maternal and early life exposure to manganese in rural Bangladesh. Environ Sci Technol.

[20] Leyva-Illades D, Chen P, Zogzas CE, Hutchens S, Mercado JM, Swaim CD, Morrisett RA, Bowman AB, Aschner M, Mukhopadhyay S (2014). SLC30A10 is a cell surface-localized manganese efflux transporter, and parkinsonism-causing mutations block its intracellular trafficking and efflux activity. J Neurosci.

[21] Tuschl K, Meyer E, Valdivia LE, Zhao N, Dadswell C, Abdul-Sada A, Hung CY, Simpson MA, Chong WK, Jacques TS, Woltjer RL, Eaton S, Gregory A, Sanford L, Kara E, Houlden H, Cuno SM, Prokisch H, Valletta L, Tiranti V, Younis R, Maher ER, Spencer J, Straatman-Iwanowska A, Gissen P, Selim LA, Pintos-Morell G, Coroleu-Lletget W, Mohammad SS, Yoganathan S, Dale RC, Thomas M, Rihel J, Bodamer OA, Enns CA, Hayflick SJ, Clayton PT, Mills PB, Kurian MA, Wilson SW (2016). Mutations in SLC39A14 disrupt manganese homeostasis and cause childhood-onset parkinsonism-dystonia. Nat Commun..

[22] Quadri M, Federico A, Zhao T, Breedveld GJ, Battisti C, Delnooz C (2012). Mutations in SLC30A10 cause parkinsonism and dystonia with hypermanganesemia, polycythemia, and chronic liver disease. Am J Hum Genet..

[23] Tuschl K, Clayton PT, Gospejr SM, Gulab S, Ibrahim S, Singhi P (2012). Syndrome of hepatic cirrhosis, dystonia, polycythemia, and hypermanganesemia caused by mutations in SLC30A10, a manganese transporter in man. Am J Hum Genet..

[24] Park JH, Hogrebe M, Grüneberg M, DuChesne I, von der Heiden AL, Reunert J, Schlingmann KP, Boycott KM, Beaulieu CL, Mhanni AA, Innes AM, Hörtnagel K, Biskup S, Gleixner EM, Kurlemann G, Fiedler B, Omran H, Rutsch F, Wada Y, Tsiakas K, Santer R, Nebert DW, Rust S, Marquardt T (2015). SLC39A8 deficiency: a disorder of manganese transport and glycosylation. Am J Hum Genet.

[25] Ng E, Lind PM, Lindgren C, Ingelsson E, Mahajan A, Morris A, Lind L (2015). Genome-wide association study of toxic metals and trace elements reveals novel associations. Hum Mol Genet.

[26] Walberg K, Kippler M, Alhamdow A, Rahman SM, Smith DR, Vahter M, Lucchini R, Broberg K (2015). Common polymorphisms in the solute carrier SLC30A10 are associated with blood manganese and neurological function. Toxicol Sci.

[27] Wahlberg K, Arora M, Curtin A, Curtin P, Wright RO, Smith DR, Lucchini RG, Broberg K, Austin C (2017). Polymorphisms in manganese transporters show developmental stage and sex specific associations with manganese concentrations in primary teeth. Neurotoxicology.

[28] Wahlberg KE, Guazzetti S, Pineda D, Larsson SC, Fedrighi C, Cagna G, Zoni S, Placidi D, Wright RO, Smith DR, Lucchini RG, Broberg K (2018). Polymorphisms in manganese transporters SLC30A10 and SLC39A8 are associated with children’s neurodevelopment by influencing manganese homeostasis. Front Gen.

[29] Gao J, Tong L, Argos M, Scannell Bryan M, Ahmed A, Rakibuz-Zaman M, Kibriya MG, Jasmine F, Slavkovich V, Graziano JH, Ahsan H, Pierce BL (2015). The genetic architecture of arsenic metabolism efficiency: a SNP-based heritability study of Bangladeshi adults. Environ Health Perspect.

[30] Broberg K, Taj T, Guazzetti S, Peli M, Cagna G, Pineda D, Placidi D, Wright RO, Smith DR, Lucchini RG, Wahlberg K (2019). Manganese transporter genetics and sex modify the association between environmental manganese exposure and neurobehavioral outcomes in children. Environment International.

[31] Leyva-Illades D, Chen P, Zogzas CE, Hutchens S, Mercado JM, Swaim CD, Morrisett RA, Bowman AB, Aschner M, Mukhopadhyay S (2014). SLC30A10 is a cell surface-localized manganese efflux transporter, and parkinsonism-causing mutations block its intracellular trafficking and efflux activity. J Neurosci.

[32] Boycott KM, Beaulieu CL, Kernohan KD, Gebril OH, Mhanni A, Chudley AE, Redl D, Qin W, Hampson S, Küry S, Tetreault M, Puffenberger EG, Scott JN, Bezieau S, Reis A, Uebe S, Schumacher J, Hegele RA, McLeod DR, Gálvez-Peralta M, Majewski J, Ramaekers VT, Nebert DW, Innes AM, Parboosingh JS, Abou JR, Care4Rare Canada Consortium (2015). Autosomal-recessive intellectual disability with cerebellar atrophy syndrome caused by mutation of the manganese and zinc transporter gene SLC39A8. Am J Hum Genet..

[33] Ye Q, Kim J (2016). Mutation in HFE gene decreases manganese accumulation and oxidative stress in the brain after olfactory manganese exposure. Metallomics.

[34] Fleming RE, Sly WS (2002). Mechanisms of iron accumulation in hereditary hemochromatosis. Annu Rev Physiol..

[35] Claus Henn B, Kim J, Wessling-Resnick M, Téllez-Rojo MM, Jayawardene I, Ettinger AS, Hernández-Avila M, Schwartz J, Christiani DC, Hu H, Wright RO (2011). Associations of iron metabolism genes with blood manganese levels: a population-based study with validation data from animal models. Environ Health..

[36] Van Landeghem GF, Sikström C, Beckman LE, Adolfsson R, Beckman L (1998). Transferrin C2, metal binding and Alzheimer’s disease. NeuroReport.

[37] Almeida QJ, Wishart LR, Lee TD (2002). Bimanual coordination deficits with Parkinson’s disease: the influence of movement speed and external cueing. Mov Disord.

[38] Rentschler G, Covolo L, Haddad A, Lucchini R, Zoni S, Broberg K (2012). ATP13A2 (PARK9) polymorphisms influence the neurotoxic effects of manganese. Neurotoxicology.

[39] Hao Y, Yan L, Pang Y, Yan H, Zhang L, Liu J, Li N, Wang B, Zhang Y, Li Z, Ye R, Ren A (2020). Maternal serum level of manganese, single nucleotide polymorphisms, and risk of spontaneous preterm birth: a nested case-control study in China. Environ Pollut..

[40] Rahbar MH, Samms-Vaughan M, Saroukhani S, Lee M, Zhang J, Bressler J, Hessabi M, Shakespeare-Pellington S, Grove ML, Loveland KA (2021). Interaction of blood manganese concentrations with GSTT1 in relation to autism spectrum disorder in Jamaican children. J Autism Dev Disord.

[41] Piacentini S, Polimanti R, Porreca F, Martínez-Labarga C, De Stefano GF, Fuciarelli M (2011). GSTT1 and GSTM1 gene polymorphisms in European and African populations. Mol Biol Rep.

[42] Giau VV, Bagyinszky E, An SS, Kim SY (2015). Role of apolipoprotein E in neurodegenerative diseases. Neuropsychiatr Dis Treat.

[43] Trdin A, Snoj Tratnik J, Stajnko A, Marc J, Mazej D, Sešek Briški A, Kastelec D, Prpić I, Petrović O, Špirić Z, Horvat M, Falnoga I (2020). Trace elements and APOE polymorphisms in pregnant women and their new-borns. Environ Int..

[44] Sunuwar L, Frkatović A, Sharapov S, Wang Q, Neu HM, Wu X, Haritunians T, Wan F, Michel S, Wu S, Donowitz M, McGovern D, Lauc G, Sears C, Melia J (2020). Pleiotropic ZIP8 A391T implicates abnormal manganese homeostasis in complex human disease. JCI Insight.

[45] Speliotes EK, Willer CJ, Berndt SI, Monda KL, Thorleifsson G, Jackson AU, Lango Allen H, Lindgren CM, Luan J, Mägi R, Randall JC, Vedantam S, Winkler TW, Qi L, Workalemahu T, Heid IM, Steinthorsdottir V, Stringham HM, Weedon MN, Wheeler E, Wood AR, Ferreira T, Weyant RJ, Segrè AV, Estrada K, Liang L, Nemesh J, Park JH, Gustafsson S, Kilpeläinen TO, Yang J, Bouatia-Naji N, Esko T, Feitosa MF, Kutalik Z, Mangino M, Raychaudhuri S, Scherag A, Smith AV, Welch R, Zhao JH, Aben KK, Absher DM, Amin N, Dixon AL, Fisher E, Glazer NL, Goddard ME, Heard-Costa NL, Hoesel V, Hottenga JJ, Johansson A, Johnson T, Ketkar S, Lamina C, Li S, Moffatt MF, Myers RH, Narisu N, Perry JR, Peters MJ, Preuss M, Ripatti S, Rivadeneira F, Sandholt C, Scott LJ, Timpson NJ, Tyrer JP, van Wingerden S, Watanabe RM, White CC, Wiklund F, Barlassina C, Chasman DI, Cooper MN, Jansson JO, Lawrence RW, Pellikka N, Prokopenko I, Shi J, Thiering E, Alavere H, Alibrandi MT, Almgren P, Arnold AM, Aspelund T, Atwood LD, Balkau B, Balmforth AJ, Bennett AJ, Ben-Shlomo Y, Bergman RN, Bergmann S, Biebermann H, Blakemore AI, Boes T, Bonnycastle LL, Bornstein SR, Brown MJ, Buchanan TA, Busonero F, Campbell H, Cappuccio FP, Cavalcanti-Proença C, Chen YD, Chen CM, Chines PS, Clarke R, Coin L, Connell J, Day IN, den Heijer M, Duan J, Ebrahim S, Elliott P, Elosua R, Eiriksdottir G, Erdos MR, Eriksson JG, Facheris MF, Felix SB, Fischer-Posovszky P, Folsom AR, Friedrich N, Freimer NB, Fu M, Gaget S, Gejman PV, Geus EJ, Gieger C, Gjesing AP, Goel A, Goyette P, Grallert H, Grässler J, Greenawalt DM, Groves CJ, Gudnason V, Guiducci C, Hartikainen AL, Hassanali N, Hall AS, Havulinna AS, Hayward C, Heath AC, Hengstenberg C, Hicks AA, Hinney A, Hofman A, Homuth G, Hui J, Igl W, Iribarren C, Isomaa B, Jacobs KB, Jarick I, Jewell E, John U, Jørgensen T, Jousilahti P, Jula A, Kaakinen M, Kajantie E, Kaplan LM, Kathiresan S, Kettunen J, Kinnunen L, Knowles JW, Kolcic I, König IR, Koskinen S, Kovacs P, Kuusisto J, Kraft P, Kvaløy K, Laitinen J, Lantieri O, Lanzani C, Launer LJ, Lecoeur C, Lehtimäki T, Lettre G, Liu J, Lokki ML, Lorentzon M, Luben RN, Ludwig B; MAGIC, Manunta P, Marek D, Marre M, Martin NG, McArdle WL, McCarthy A, McKnight B, Meitinger T, Melander O, Meyre D, Midthjell K, Montgomery GW, Morken MA, Morris AP, Mulic R, Ngwa JS, Nelis M, Neville MJ, Nyholt DR, O'Donnell CJ, O'Rahilly S, Ong KK, Oostra B, Paré G, Parker AN, Perola M, Pichler I, Pietiläinen KH, Platou CG, Polasek O, Pouta A, Rafelt S, Raitakari O, Rayner NW, Ridderstråle M, Rief W, Ruokonen A, Robertson NR, Rzehak P, Salomaa V, Sanders AR, Sandhu MS, Sanna S, Saramies J, Savolainen MJ, Scherag S, Schipf S, Schreiber S, Schunkert H, Silander K, Sinisalo J, Siscovick DS, Smit JH, Soranzo N, Sovio U, Stephens J, Surakka I, Swift AJ, Tammesoo ML, Tardif JC, Teder-Laving M, Teslovich TM, Thompson JR, Thomson B, Tönjes A, Tuomi T, van Meurs JB, van Ommen GJ, Vatin V, Viikari J, Visvikis-Siest S, Vitart V, Vogel CI, Voight BF, Waite LL, Wallaschofski H, Walters GB, Widen E, Wiegand S, Wild SH, Willemsen G, Witte DR, Witteman JC, Xu J, Zhang Q, Zgaga L, Ziegler A, Zitting P, Beilby JP, Farooqi IS, Hebebrand J, Huikuri HV, James AL, Kähönen M, Levinson DF, Macciardi F, Nieminen MS, Ohlsson C, Palmer LJ, Ridker PM, Stumvoll M, Beckmann JS, Boeing H, Boerwinkle E, Boomsma DI, Caulfield MJ, Chanock SJ, Collins FS, Cupples LA, Smith GD, Erdmann J, Froguel P, Grönberg H, Gyllensten U, Hall P, Hansen T, Harris TB, Hattersley AT, Hayes RB, Heinrich J, Hu FB, Hveem K, Illig T, Jarvelin MR, Kaprio J, Karpe F, Khaw KT, Kiemeney LA, Krude H, Laakso M, Lawlor DA, Metspalu A, Munroe PB, Ouwehand WH, Pedersen O, Penninx BW, Peters A, Pramstaller PP, Quertermous T, Reinehr T, Rissanen A, Rudan I, Samani NJ, Schwarz PE, Shuldiner AR, Spector TD, Tuomilehto J, Uda M, Uitterlinden A, Valle TT, Wabitsch M, Waeber G, Wareham NJ, Watkins H; Procardis Consortium, Wilson JF, Wright AF, Zillikens MC, Chatterjee N, McCarroll SA, Purcell S, Schadt EE, Visscher PM, Assimes TL, Borecki IB, Deloukas P, Fox CS, Groop LC, Haritunians T, Hunter DJ, Kaplan RC, Mohlke KL, O'Connell JR, Peltonen L, Schlessinger D, Strachan DP, van Duijn CM, Wichmann HE, Frayling TM, Thorsteinsdottir U, Abecasis GR, Barroso I, Boehnke M, Stefansson K, North KE, McCarthy MI, Hirschhorn JN, Ingelsson E, Loos RJ. Association analyses of 249,796 individuals reveal 18 new loci associated with body mass index. Nat Genet. 2010;42(11):937–48; 10.1038/ng.686 10.1038/ng.686 PMC3014648 20935630

[46] Carrera N, Arrojo M, Sanjuán J, Ramos-Ríos R, Paz E, Suárez-Rama JJ, Páramo M, Agra S, Brenlla J, Martínez S, Rivero O, Collier DA, Palotie A, Cichon S, Nöthen MM, Rietschel M, Rujescu D, Stefansson H, Steinberg S, Sigurdsson E, St Clair D, Tosato S, Werge T, Stefansson K, González JC, Valero J, Gutiérrez-Zotes A, Labad A, Martorell L, Vilella E, Carracedo Á, Costas J (2012). Association study of nonsynonymous single nucleotide polymorphisms in schizophrenia. Biol Psychiatry.

[47] Hermann ER, Chambers E, Davis DN, Montgomery MR, Lin D, Chowanadisai W (2021). Brain magnetic resonance imaging phenome-wide association study with metal transporter gene SLC39A8. Front Genet..

[48] Haynes EN, Heckel P, Ryan P, Roda S, Leung YK, Sebastian K, Succop P (2010). Environmental manganese exposure in residents living near a ferromanganese refinery in Southeast Ohio: a pilot study. Neurotoxicology.

[49] van Veen S, Martin S, Van den Haute C, Benoy V, Lyons J, Vanhoutte R, Kahler JP, Decuypere JP, Gelders G, Lambie E, Zielich J, Swinnen JV, Annaert W, Agostinis P, Ghesquière B, Verhelst S, Baekelandt V, Eggermont J, Vangheluwe P (2020). ATP13A2 deficiency disrupts lysosomal polyamine export. Nature.

[50] Greenberg MVC, Bourc'his D (2019). The diverse roles of DNA methylation in mammalian development and disease. Nat Rev Mol Cell Biol.

[51] Ameres SL, Zamore PD (2013). Diversifying microRNA sequence and function. Nat Rev Mol Cell Biol.

[52] Morgan MAJ, Shilatifard A (2020). Reevaluating the roles of histone-modifying enzymes and their associated chromatin modifications in transcriptional regulation. Nat Genet.

[53] Gluckman PD, Hanson MA, Cooper C, Thornburg KL (2008). Effect of in utero and early-life conditions on adult health and disease. N Engl J Med.

[54] Joubert BR, Håberg SE, Nilsen RM, Wang X, Vollset SE, Murphy SK, Huang Z, Hoyo C, Midttun Ø, Cupul-Uicab LA, Ueland PM, Wu MC, Nystad W, Bell DA, Peddada SD, London SJ (2012). 450K epigenome-wide scan identifies differential DNA methylation in newborns related to maternal smoking during pregnancy. Environ Health Perspect.

[55] Cediel Ulloa A, Gliga A, Love TM, Pineda D, Mruzek DW, Watson GE, Davidson PW, Shamlaye CF, Strain JJ, Myers GJ, van Wijngaarden E, Ruegg J, Broberg K (2021). Prenatal methylmercury exposure and DNA methylation in seven-year-old children in the Seychelles Child Development Study. Environ Int..

[56] Smeester L, Fry RC (2018). Long-term health effects and underlying biological mechanisms of developmental exposure to arsenic. Curr Environ Health Rep.

[57] Rodan LH, Hauptman M, D'Gama AM, Qualls AE, Cao S, Tuschl K, Al-Jasmi F, Hertecant J, Hayflick SJ, Wessling-Resnick M, Yang ET, Berry GT, Gropman A, Woolf AD, Agrawal PB (2018). Novel founder intronic variant in SLC39A14 in two families causing manganism and potential treatment strategies. Mol Genet Metab.

[58] Stamelou M, Tuschl K, Chong WK, Burroughs AK, Mills PB, Bhatia KP, Clayton PT (2012). Dystonia with brain manganese accumulation resulting from SLC30A10 mutations: a new treatable disorder. Mov Disord.

[59] Cantone L, Nordio F, Hou L, Apostoli P, Bonzini M, Tarantini L, Angelici L, Bollati V, Zanobetti A, Schwartz J, Bertazzi PA, Baccarelli A (2011). Inhalable metal-rich air particles and histone H3K4 dimethylation and H3K9 acetylation in a cross-sectional study of steel workers. Environ Health Perspect.

[60] Nwanaji-Enwerem JC, Colicino E, Specht AJ, Gao X, Wang C, Vokonas P, Weisskopf MG, Boyer EW, Baccarelli AA, Schwartz J (2020). Individual species and cumulative mixture relationships of 24-hour urine metal concentrations with DNA methylation age variables in older men. Environ Res..

[61] Kresovich JK, Bulka CM, Joyce BT, Vokonas PS, Schwartz J, Baccarelli AA, Hibler EA, Hou L (2018). The inflammatory potential of dietary manganese in a cohort of elderly men. Biol Trace Elem Res.

[62] Bozack AK, Rifas-Shiman SL, Coull BA, Baccarelli AA, Wright RO, Amarasiriwardena C, Gold DR, Oken E, Hivert MF, Cardenas A (2021). Prenatal metal exposure, cord blood DNA methylation and persistence in childhood: an epigenome-wide association study of 12 metals. Clin Epigenetics.

[63] Aung MT, Bakulski KM, Feinberg JI, Dou JF, Meeker JD, Mukherjee B, Loch-Caruso R, Ladd-Acosta C, Volk HE, Croen LA, Hertz-Picciotto I, Newschaffer CJ, Fallin MD (2021). Maternal blood metal concentrations and whole blood DNA methylation during pregnancy in the Early Autism Risk Longitudinal Investigation (EARLI). Epigenetics..

[64] Maccani JZ, Koestler DC, Houseman EA, Armstrong DA, Marsit CJ, Kelsey KT (2015). DNA methylation changes in the placenta are associated with fetal manganese exposure. Reprod Toxicol..

[65] Appleton AA, Jackson BP, Karagas M, Marsit CJ (2017). Prenatal exposure to neurotoxic metals is associated with increased placental glucocorticoid receptor DNA methylation. Epigenetics..

[66] Gutiérrez-González E, García-Esquinas E, de Larrea-Baz NF, Salcedo-Bellido I, Navas-Acien A, Lope V, Gómez-Ariza JL, Pastor R, Pollán M, Pérez-Gómez B (2019). Toenails as biomarker of exposure to essential trace metals: a review. Environ Res.

[67] Holmes L, Shutman E, Chinaka C, Deepika K, Pelaez L, Dabney KW (2019). Aberrant epigenomic modulation of glucocorticoid receptor gene (NR3C1) in early life stress and major depressive disorder correlation: systematic review and quantitative evidence synthesis. Int J Environ Res Public Health.

[68] Wang L, Shiraki A, Itahashi M, Akane H, Abe H, Mitsumori K (2013). Aberration in epigenetic gene regulation in hippocampal neurogenesis by developmental exposure to manganese chloride in mice. Toxicol Sci.

[69] Hill DS, Cabrera R, Wallis Schultz D, Zhu H, Lu W, Finnell RH, Wlodarczyk BJ (2015). Autism-like behavior and epigenetic changes associated with autism as consequences of in utero exposure to environmental pollutants in a mouse model. Behav Neurol..

